# Targeting regulation of stem cell exosomes: Exploring novel strategies for aseptic loosening of joint prosthesis

**DOI:** 10.3389/fbioe.2022.925841

**Published:** 2022-08-10

**Authors:** Tian-Liang Ma, Jing-Xian Chen, Zhuo-Ran Ke, Peng Zhu, Yi-He Hu, Jie Xie

**Affiliations:** ^1^ Department of Orthopedics, Xiangya Hospital, Central South University, Changsha, China; ^2^ Hunan Engineering Research Center of Biomedical Metal and Ceramic Impants, Xiangya Hospital, Central South University, Changsha, China; ^3^ XiangYa School of Medicine, Central South University, Changsha, China

**Keywords:** stem cell exosomes, joint replacement, aseptic loosening, bone metabolism, anti-inflammatory

## Abstract

Periprosthetic osteolysis is a major long-term complication of total joint replacement. A series of biological reactions caused by the interaction of wear particles at the prosthesis bone interface and surrounding bone tissue cells after artificial joint replacement are vital reasons for aseptic loosening. Disorder of bone metabolism and aseptic inflammation induced by wear particles are involved in the occurrence and development of aseptic loosening of the prosthesis. Promoting osteogenesis and angiogenesis and mediating osteoclasts and inflammation may be beneficial in preventing the aseptic loosening of the prosthesis. Current research about the prevention and treatment of aseptic loosening of the prosthesis focuses on drug, gene, and stem cell therapy and has not yet achieved satisfactory clinical efficacy or has not been used in clinical practice. Exosomes are a kind of typical extracellular vehicle. In recent years, stem cell exosomes (Exos) have been widely used to regulate bone metabolism, block inflammation, and have broad application prospects in tissue repair and cell therapy.

## Introduction

Arthroplasty is currently the treatment of choice for terminal osteoarticular diseases and is the mainstay of treating joint diseases, relieving joint pain, and reconstructing joint function (Rachner et al., 2011; Beckmann et al., 2021; Szczesiul and Bielecki, 2021). Among them, revision surgery accounts for a significant proportion of joint replacements, and one of the significant reasons lies in aseptic loosening caused by particulate wear debris around the prosthesis (Rachner et al., 2011; Hampton et al., 2020; Hodges et al., 2021). Searching for exosome and aseptic loosening in PubMed, only three results could be obtained. Therefore, bone metabolism, angiogenesis, and aseptic loosening were searched, and 481 and nine results were obtained. The articles and reviewers with high credibility in recent years were selected from 481 results, and the contents were sorted out to obtain this article. Aseptic loosening mainly involves macrophages, osteoblasts, and osteoclasts. Macrophages release a series of pro-inflammatory factors, such as tumor necrosis factor-α (TNF-α), interleukin-1 (IL-1), and IL-23. After recognizing and phagocytosing wear particles, dysregulation of the receptor activator of nuclear factor κB-receptor activator of nuclear factor κB (NF-κB) ligand-osteoclastogenesis inhibitory factor (RANK-RANKL-OPG) axis is caused. For example, TNF-α and IL-1 stimulate osteoblasts to express RANKL; IL-23 promotes the differentiation of CD4^+^ T cells into the T helper cell 17 (Th17) phenotype, and IL-17 secreted by Th17 cells is a potent inducer of RANKL expression (Lubberts et al., 2003; Lin et al., 2017b). The RANK–RANKL–OPG signaling pathway is a vital pathway regulating osteoclast formation, activation, and survival (Boyce and Xing, 2007). RANK is expressed by osteoclast precursor cells and mature osteoclasts. Activation of RANK promotes the RANK-mediated NF-κB signaling pathway, which in turn encourages osteoclastogenesis and activates osteoclasts (Rachner et al., 2011; Nagy and Penninger, 2015). RANKL is a ligand for RANK that activates RANK on the surface of osteoclast precursor cells, causing a series of activations such as tumor necrosis factor receptor–associated factor 6 (TRAF6), mitogen-activated protein kinases (MAPKs), and transcription factors NF-κB and activator protein-1 (AP-1), which in turn promote the differentiation, activation, and survival of osteoclasts (Boyce and Xing, 2007; Altaf and Revell, 2013; Park et al., 2017). OPG, a decoy receptor that binds to RANKL and inhibits RANK–RANKL interaction, is expressed by vascular endothelial cells and fibroblasts in periprosthetic tissues and inhibits osteoclast activation (Crotti et al., 2004; Koreny et al., 2006). In aseptic loosening of the prosthesis, the expression of OPG is downregulated, and the RANKL/OPG ratio is activated, indicating enhanced osteolysis at the time of OPG decompensation (Hartmann et al., 2017). In summary, regulating bone metabolism and inhibiting inflammation after macrophages phagocytose wear particles are significant entry points for the prevention and treatment of aseptic loosening of prostheses.

Exosomes are small endogenous vesicles with a diameter of about 40–160 nm secreted by cells and contain proteins, lipids, metabolites, and nucleic acids (mRNA, non-coding RNA, and DNA). Exosomes have been reported to play a critical role in removing excess or unnecessary intracellular components and regulating intercellular communication (Kalluri and LeBleu, 2020). In recent years, stem cell exosomes have played a vital role in the treatment of osteoarticular diseases, including bone marrow–derived mesenchymal stem cells (BMSCs), adipose-derived stem cells (ADSCs), umbilical cord blood–derived mesenchymal stem cells (UCB-MSCs), and urine-derived stem cells (USCs). BMSCs are the earliest primary source of pluripotent stem cells, and their culture time is relatively short (Berebichez-Fridman et al., 2017; Berebichez-Fridman and Montero-Olvera, 2018). However, their cell yield, lifespan, and differentiation potential decrease with donor age (Kern et al., 2006; Cagliani et al., 2017; Berebichez-Fridman and Montero-Olvera, 2018). ADSCs are stem cells derived from adipose tissue. Subcutaneous adipose tissue is found throughout the body, and 98–100 percent of cells derived from adipose tissue are viable (Liras, 2010; Choudhery et al., 2014). Studies have indicated that age influences the expansion and differentiation of ADSCs, especially in osteogenic and cartilaginous lineages (Choudhery et al., 2014). UCB-MSCs are derived from the umbilical cord and are considered the most primitive cells among MSCs of various tissue origins, with easily accessible and non-invasive properties (Yang et al., 2020a). UCB-MSCs secrete more wound healing factors (such as the extracellular matrix–degrading enzymes, matrix metalloproteinase-2 and urokinase-type plasminogen activator) than other MSCs (Doi et al., 2016; Kim et al., 2017) and promote fibroblast migration, proliferation, and collagen synthesis (Luo et al., 2010). USCs are derived from fresh human urine and have the advantages of non-invasiveness, easy access, sustainable production, and the relative absence of ethical issues (Li et al., 2020). USCs also have the ability for solid proliferation, lipogenesis, endothelial differentiation, and vascularization compared with BMSCs (Wu et al., 2018).

Recent studies have demonstrated that stem cell exosomes play a crucial and essential role in the process of bone metabolism and anti-inflammation (Qin et al., 2016; Li et al., 2018; Shi et al., 2019; Yang et al., 2020a). Different stem cell exosomes can play substantial roles in enhancing osteogenesis, suppressing osteoclast activity, augmenting angiogenesis, and resisting inflammation (Tofiño-Vian et al., 2017; Shi et al., 2019). Therefore, the application of stem cell exosomes is theoretically promising as an effective intervention to prevent and treat the aseptic loosening of prostheses.

## Enhance osteogenesis and suppress osteoclast activity

Bone formation and bone resorption are central components of bone metabolism in the ternary regulation theory of bone metabolism (Marie and Kassem, 2011). Bone formation is the primary process of bone development. When bone formation is more significant than bone absorption, bone develops. When bone mass reaches its peak, bone formation and absorption are in a dynamic equilibrium stage (Buettmann et al., 2019; Zhang et al., 2020). Osteoclasts are more active than osteoblasts in a pathological state, and bone resorption exceeds bone formation. Unbalanced bone resorption and bone formation eventually result in bone loss (Wu et al., 2010). While in other pathological states, with excessive osteoblasts, unbalanced bone formation and resorption also lead to excessive bone formation rather than bone loss (Gao et al., 2018). Osteoblasts are equipped with the potential to differentiate into osteocytes (Marie and Kassem, 2011; Pajarinen et al., 2017). Osteoclasts gather around apoptotic bone cells to further recruit osteoclasts. Interestingly, communication between osteoblasts and osteoclasts occurs through EVs (Yuan et al., 2018). Wear debris triggers bone resorption by activating macrophages and osteoclasts and directly impairs bone formation by attenuating osteoblast function (Pajarinen et al., 2017). Studies have focused on periprosthetic osteolysis on osteoclasts, macrophages, and fibroblasts. Once worn debris is exposed *in vitro*, these cells release pro-inflammatory cytokines that may activate osteoclasts through multiple pathways, leading to bone loss (Kusano et al., 1998; Lader and Flanagan, 1998). In addition, osteoblasts secrete cytokines to recruit inflammatory cells into the periprosthetic space and stimulate bone resorption by osteoclasts (Vermes et al., 2001). Therefore, silencing osteoclast-mediated osteolysis around the prosthesis is of great significance for preventing aseptic loosening of the prosthesis.

Various stem cell exosomes block osteoclast activation or directly differentiate into osteoblasts to regulate bone remodeling. BMSC-Exos activates osteogenesis and downregulates osteoclastogenesis through multiple pathways. BMSC-Exos transplantation plays a key role in the treatment of osteoporosis by promoting osteogenesis, which is attributed to the activation of bone morphogenetic protein-2-drosophila mothers against decapentaplegic protein1runt-related transcription factor-2 (BMP-2/Smad1/RUNX2) and hypoxia-inducible factor-1-vascular endothelial growth factor (HIF-1α/VEGF) signaling pathways (Zhang et al., 2020). BMSC-Exos contribute to bone healing during fracture healing by carrying miR-126 and alleviate radiation-induced bone loss by activating the Wnt/β-catenin pathway (Lu et al., 2020). In aged BMSCs, the expression level of miR-31a-5p was higher, which leads to adipogenesis and cell senescence and attenuates cell osteogenesis (Xu et al., 2018). In addition, exosomes secreted by pre-differentiated human mesenchymal stem cells (hMSCs) for a certain period induce osteogenic differentiation, including upregulating osteogenic miRNA (Hsa-miR-146a-5p, Hsa-miR-503-5p, Hsa-miR-483-3p, and Hsa-miR-129-5p) and downregulating anti-osteogenic miRNA (Hsa-miR-32-5p, Hsa-miR-133a-3p, and Hsa-miR-204-5p) to activate phosphoinositol-3-kinase-protein kinase B (PI3K/Akt) and MAPK signaling pathways. hMSC exosomes are used as inducers to induce osteogenic differentiation of hMSCs *in vitro* (Zhai et al., 2020). Pathologically, BMSC-Exos extracted from patients with osteoporosis attenuate osteogenesis by downregulating SMAD7 *via* miR-21 (Jiang, Tian, Zhang). In summary, BMSC-Exos have obvious bone-promoting and bone-suppressing effects under physiological conditions. The treatment centered on BMSC-Exos is expected to become a strategy for clinical prevention of aseptic loosening of the prosthesis.

Adipose-derived, stem-cell-derived exosomes (ADSC-Exos) have a decent osteogenic effect. It was found that the overexpression of miR-130a-3p, the exosome of ADSCs, could enhance osteogenic differentiation of ADSCs and reduce the protein and mRNA levels of silent information regulator 7 (SIRT7), the target of miR-130a-3p. Overexpression of miR-130a-3p resulted in downregulation of SIRT7 and upregulation of Wnt signaling pathway–related proteins, suggesting that exosome miR-130a-3p upregulates osteogenic differentiation of ADSCs by partially mediating the SIRT7/Wnt/β-catenin axis (Yang et al., 2020b). ADSC-Exos decreased RANKL expression at mRNA and protein levels and decreased RANKL/OPG ratio at the gene level. ADSC-Exos antagonized hypoxia and serum deprivation–induced osteocyte apoptosis and osteoclastogenesis (Ren et al., 2019). Transplantation of UCB-MSCs derived from human umbilical cord blood or its conditioned medium prevents bone loss in ovariectomized nude mice, drastically enhances the survival rate of bone-like MLO-Y4 cells, and mediates osteoclast differentiation. *In vitro*, the conditional medium (CM) of UCB-MSCs activates alkaline phosphatase (ALP) in human BMSCs and the mRNA expression of type 1 collagen, RUNX2, osterix, and ALP in C3H10T1/2 cells, indicating that it had apparent osteogenic activity (An et al., 2013), which was mediated by a paracrine mechanism.

USCs also have more vigorous proliferation and stronger abiogenesis, endothelial differentiation, and vascularization than BMSCs (Wu et al., 2018). Autologous USC-Exos are promising osteoporosis therapeutic agents that enrich osteogenesis and block osteoclastogenesis by transporting silent information regulator 7 (CTHRC1) and OPG (Chen et al., 2019). In addition, our previous research results show that USC-Exos effectively cause osteogenic differentiation and generation and attenuate osteoclast differentiation.

**FIGURE 1:**
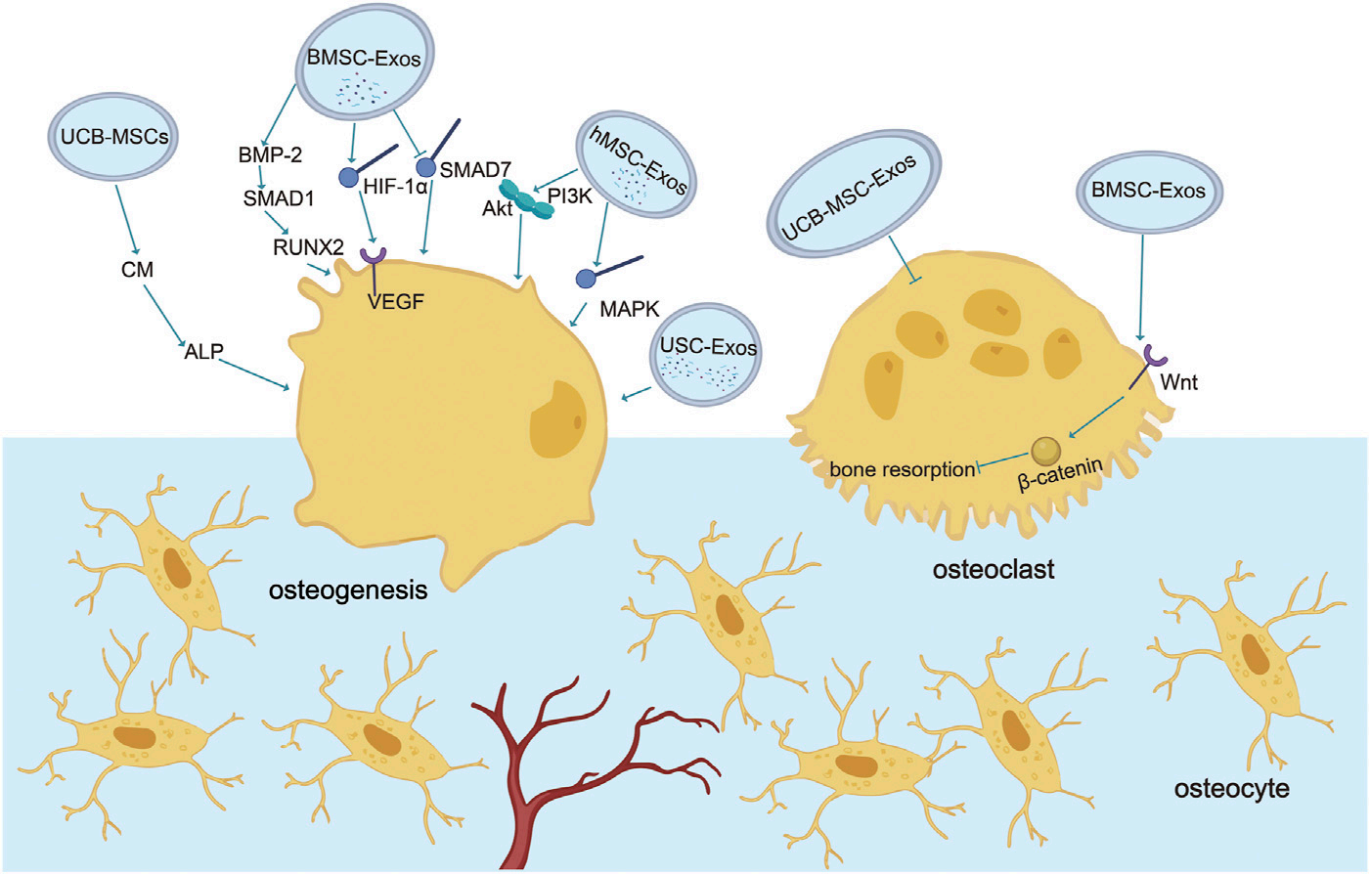
Role of exosomes in osteogenesis and osteoclasts. Various stem cell exosomes block osteoclast activation or directly differentiate into osteoblasts to regulate bone remodeling. Communication between osteoblasts and osteoclasts occurs through EVs. BMSC-Exos activate osteogenesis through BMP-2-SMAD1-RUNX2, HIF-1α-VEGF, and SMAD7 pathways and block osteoclastogenesis through the classic Wnt-β-catenin pathway. UCB-Exos activate ALP in human BMSC and the mRNA expression of type 1 collagen, RUNX2, osterix, and ALP in C3H10T1/2 cells. USC-Exos enrich osteogenesis and block osteoclastogenesis by transporting CTHRC1 and OPG. hMSC-Exos promote osteogenesis by PI3K/Akt and MAPK.

In summary, wear debris downregulates bone formation by activating macrophages and osteoclasts to trigger bone resorption or silencing osteoblast function. At the same time, a variety of stem cell exosomes play an essential regulatory role in promoting osteogenesis and blocking osteoclasts. Therefore, targeted regulation of stem cell exosomes is expected to play a preventive and therapeutic role in the process of aseptic loosening of the prosthesis (Figure 1) (Table 1).

**TABLE 1:** Main exosomes in bone metabolism.

Exosome species	Key target	Reference
BMSC-Exos	BMP-2/Smad1/RUNX	Zhang et al. (2020)
	HIF-1α/VEGF	
	miR-126-Wnt/β-catenin	Lu et al. (2020)
	miR-31a-5p	Xu et al. (2018)
	miR-2148	(Jiang, Tian, Zhang)
	ALP, type 1 collagen, RUNX2, and osterix	An et al. (2013)
hMSC-Exos	Hsa-miR-146a-5p, Hsa-miR-503-5p, Hsa-miR-483-3p, and Hsa-miR-129-5p	Zhai et al. (2020)
	Hsa-miR-32-5p, Hsa-miR-133a-3p, and Hsa-miR-204-5p	
	PI3K/Akt	
	MAPK	
ADSC-Exos	miR-130a-3p	Yang et al. (2020b)
	SIRT7/Wnt/β-catenin	
	RANKL	Ren et al. (2019)
USC-Exos	CTHRC1 and OPG	Chen et al. (2019)

## Augment angiogenesis

Angiogenesis is the process of generating new blood vessels from the original blood vessels (Olsen et al., 2017). In 2014, nature reported a new capillary subtype in the murine skeletal system with distinct morphological, molecular, and functional properties (Kusumbe et al., 2014). These vessels are found in specific locations, mediate growth of the bone vasculature, generate distinct metabolic and molecular microenvironments, maintain perivascular osteoprogenitors, and couple angiogenesis to osteogenesis (Kusumbe et al., 2014). Vessels not only mediate the circulation of cells, oxygen, nutrients, and waste, especially the wear particles of the prosthesis, but also provide vascular secretion signals that control organ growth and homeostasis (Red-Horse et al., 2007; Butler et al., 2010; Tashiro et al., 2012). Local blood supply or angiogenesis plays a vital role in bone metabolism (Wang et al., 2017) and forms a network with surrounding bone tissue to further regulate bone metabolism. During bone development, homeostasis, and repair, dense vascular systems provide oxygen and nutrients to highly anabolic bone cells (Wang et al., 2007; Hu and Olsen, 2016). New blood vessels provide sources of circulating factors, such as parathyroid hormone and vitamin D, which are essential for the stability of the bone environment (Hankenson et al., 2011).

After joint replacement, no wear debris around the prosthesis attenuates osteoblast function, impairs bone formation, and blocks angiogenesis (Pajarinen et al., 2017). MAO-650 is a coating of microporous TiO_2_ decorated with hydroxyapatite (HA) nanoparticles. MAO-650 supports the proliferation and differentiation of osteoblasts and endothelial cells, mediates macrophage inflammatory response, and triggers favorable bone immune regulation to function as a positive regulator of bone/vascular formation and prevent aseptic loosening of prosthesis (Bai et al., 2018). Therefore, reducing the inhibitory effect of wear debris around prosthesis on angiogenesis may be an effective means for clinical prevention of aseptic loosening.

MicroRNAs in stem cell exosomes play a crucial role in angiogenesis spinal cord injury (SCI) mouse MSC loading miR-126 into exosomes. Exosomes derived from miR-126-modified MSCs contribute to human umbilical vein endothelial cell (HUVEC)–related angiogenesis and neurogenesis and attenuate apoptosis by mediating the expression of Sprouty-related EVH1 domain protein 1 (SPRED1) and phosphoinositide 3 (Huang et al., 2020). The level of miR-29a in BMSC-Exos derived from bone marrow mesenchymal stem cells is high, which is transported to HUVECs to restore angiogenesis sensitivity. Angio-inhibitory protein 1 (VASH1) was identified as a direct target of miR-29a, mediating miR-29a in BMSC-Exos to activate angiogenesis (Lu et al., 2020). Human ADSCs contribute to angiogenesis by activating the PKA signaling pathway and promoting VEGF expression. This result is used to find safe and effective treatments for traumatic diseases (Xue et al., 2018). UCB-MSC-derived exosomes reduce cisplatin-induced renal oxidative stress and apoptosis *in vivo*, increase the proliferation of cultured renal epithelial cells, promote angiogenesis, and regenerate damaged kidneys (Dorronsoro and Robbins, 2013; Xue et al., 2018). In addition, exosomes derived from UCB-MSCs contribute to injury repair. UCB-MSC-derived exosomes are encapsulated in new nanogels and injected into the sheath of the spinal cord model. The number, volume fraction, and connectivity of blood vessels in the spinal cord are dramatically raised, which regulates diabetic wounds (Zhang, Zhang, Gao, Chang, Chen, Mei, et al.). Over-metastasis of malignant brain tumor 1 (DMBT1) protein in USC-Exos causes angiogenesis, providing a new prospect for diabetic soft tissue wound healing (Chen et al., 2018). Umbilical cord mesenchymal stem cell–derived exosomes combined with Pluronic F127 hydrogel enhance granulation tissue regeneration and upregulate VEGF and transform growth factor-β1 (TGF-β1) to trigger wound healing and complete skin regeneration in chronic diabetes mellitus (Yang et al., 2020c).

**FIGURE 2:**
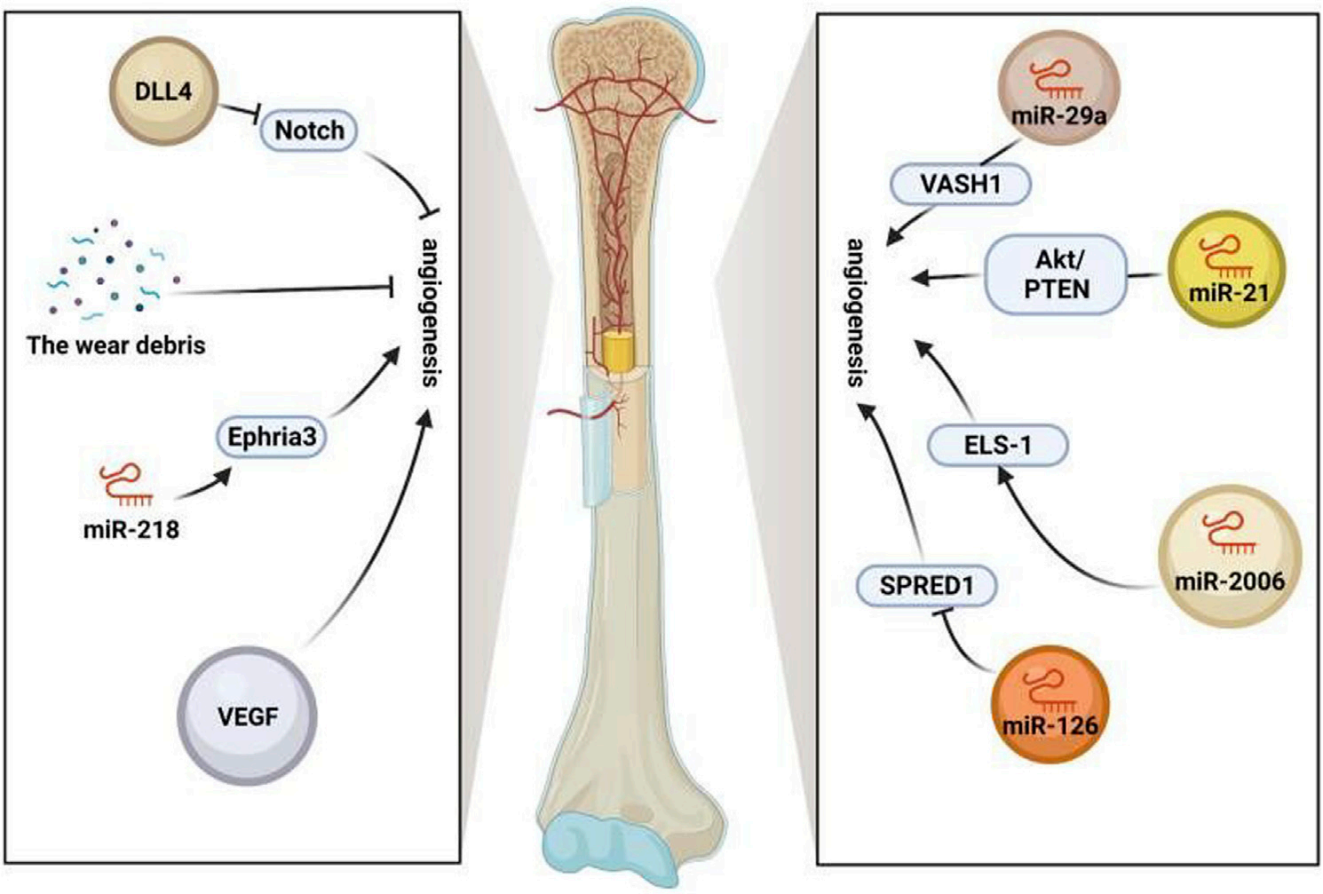
Role of exosomes in vessels. Stem cell exosomes play various vital roles in the aseptic loosening of prosthesis, including hematopoietic stem cells supporting perivascular niches and repairing and regenerating damaged bone, cartilage, and vascular tissue. Angiogenesis is promoted by VEGF, miR-218-Ephria3, miR-29a-VASH1, miR-21-PTEN/Akt, and miR-2006-ELS-1 while inhibited by the wear debris, miR-126-SPRED1, and notch (https://app.biorender.com/).

In summary, stem cell exosomes play various vital roles in the aseptic loosening of the prosthesis, including hematopoietic stem cells supporting perivascular niches and repairing and regenerating damaged bone, cartilage, and vascular tissue (Pajarinen et al., 2017; Saribas et al., 2020). Promoting angiogenesis is beneficial for providing more nutrients, metabolizing, transporting worn particles, and maintaining bone metabolism homeostasis (Red-Horse et al., 2007; Butler et al., 2010; Tashiro et al., 2012). Therefore, the use of stem cell exosomes to enhance angiogenesis is conducive to preventing the aseptic loosening of the prosthesis (Figure 2).

## Regulate immune cells and cytokines

Wear particle–induced aseptic inflammation is the leading cause of aseptic loosening of prostheses after joint replacement, so inhibition of inflammation may be a viable clinical alternative for preventing aseptic loosening of prostheses (Rachner et al., 2011; Hodges et al., 2021). Several studies have revealed that stem cell exosomes have the ability to resist inflammation mainly by regulating immune cells (macrophages, T cells, and B cells) and the cytokines they secrete.

For macrophages, stem cell exosomes play a role in anti-inflammation by inducing M2 macrophage polarization. During wound healing, after macrophages took up BMSC-Exos, exosomes induced M2 macrophage polarization through miR-223, resulting in higher IL-10 levels and decreased TNF-α levels, as shown by accelerated wound healing (He et al., 2019). In the bronchopulmonary dysplasia (BPD) model, after uptake of BMSC-Exos by alveolar macrophages, the expression levels of pro-inflammatory factors secreted by M1 macrophages such as TNF-α, IL-6, and CCL5 were blocked, and the expression levels of anti-inflammatory factors secreted by M2 macrophages such as arginase-1 (Arg-1) were increased, that is, macrophages transformed from M1 to M2, and the process occurred in a dose-dependent manner (Willis et al., 2018). In a cutaneous wound model in streptozotocin-induced diabetic rats, the induction of LPS-preconditioned umbilical cord stem-cell-derived exosomes (LPS pre-UCMSC-Exos) of M1 macrophages was sharply reduced. In contrast, the density and distribution of M2 macrophages were significantly increased. THP-1 cells produced more anti-inflammation cytokines (IL-10 and TGF-β) and M2 macrophage surface marker CD163 and fewer pro-inflammatory cytokines (IL-1, IL-6, and TNF-α). Taken together, LPS pre-UCMSC-Exos facilitated the differentiation of macrophages to M2, but not M1^70^. When peripheral blood mononuclear cells (PBMCs) were cocultured with ADSC-Exos, the mRNA expression levels of M2 macrophage markers (CD163 and Arg1) in PBMCs and the percentage of CD206 (a specific M2 macrophage marker)-positive cells were significantly increased. Moreover, M2 macrophage-specific transcription factors signal transducer and activator of transcription 6 (Stat6) and MAF BZIP transcription factor B (MafB) were activated considerably, indicating that ADSC-Exos induce the M2 phenotype of PBMCs and play a vital role in anti-inflammation (Heo et al., 2019).

For T cells, stem cell exosomes play a role in anti-inflammation by upregulating the expression of pro-inflammatory or anti-inflammation cytokines, regulating the differentiation of T cells, and inhibiting the proliferation of PBMCs. After treatment of PBMCs with BMSC-Exos, the expression levels of pro-inflammatory cytokines TNF-α and IL-1β decreased, and the expression levels of anti-inflammation cytokines TGF-β increased. The ability of exosome-induced Th1 cells to transform into Th2 cells reduced the differentiation of T cells into Th17 and reduced the production of IL-17. The expression of CTLA-4 in Treg cells emerged, which could inhibit the immune response by competing with CD28 for ligands CD80 and CD86 and then played a role in anti-inflammation (Chen, Huang, Han, Yu, Li, Lu, et al.). Treatment of PBMCs with UCB-MSC-Exos obtained by treatment with TGF-β or IFN-γ or a combination of both (MSCs-T/I) inhibited the proliferation of PBMCs, which became more pronounced with increasing dose. After treatment with MSCs-T/I exosomes, the proportion of PBMCs that transformed into Treg cells increased, and the expression of IL-10, IDO, and other anti-inflammation factors also increased, so the immunosuppressive effect and the anti-inflammation effect were enhanced (Zhang et al., 2018). In T1DM mice treated with ADSC-Exos, the number of Treg cells was significantly increased, and the levels of IL-4, IL-10, TGF-β, and other anti-inflammation factors were improved considerably. In contrast, the levels of IFN-γ, IL-17, and other pro-inflammatory factors were significantly decreased, showing a significant anti-inflammation effect (Nojehdehi et al., 2018).

For B cells, after treatment of PBMCs with BMSC-Exos, the expression levels of CXCL8 (IL-8) and marginal zone B- and B1-cell-specific protein (MZB1) were higher. The increased CXCL8 could inhibit T-cell activation and proliferation through myeloid-derived suppressor cells (MDSCs), and the increased MZB1 could cause significant inhibition of B-cell proliferation by regulating Ca^2+^. In conclusion, exosomes play a role in anti-inflammation by reducing the number and function of immune cells (Khare et al., 2018).

**FIGURE 3:**
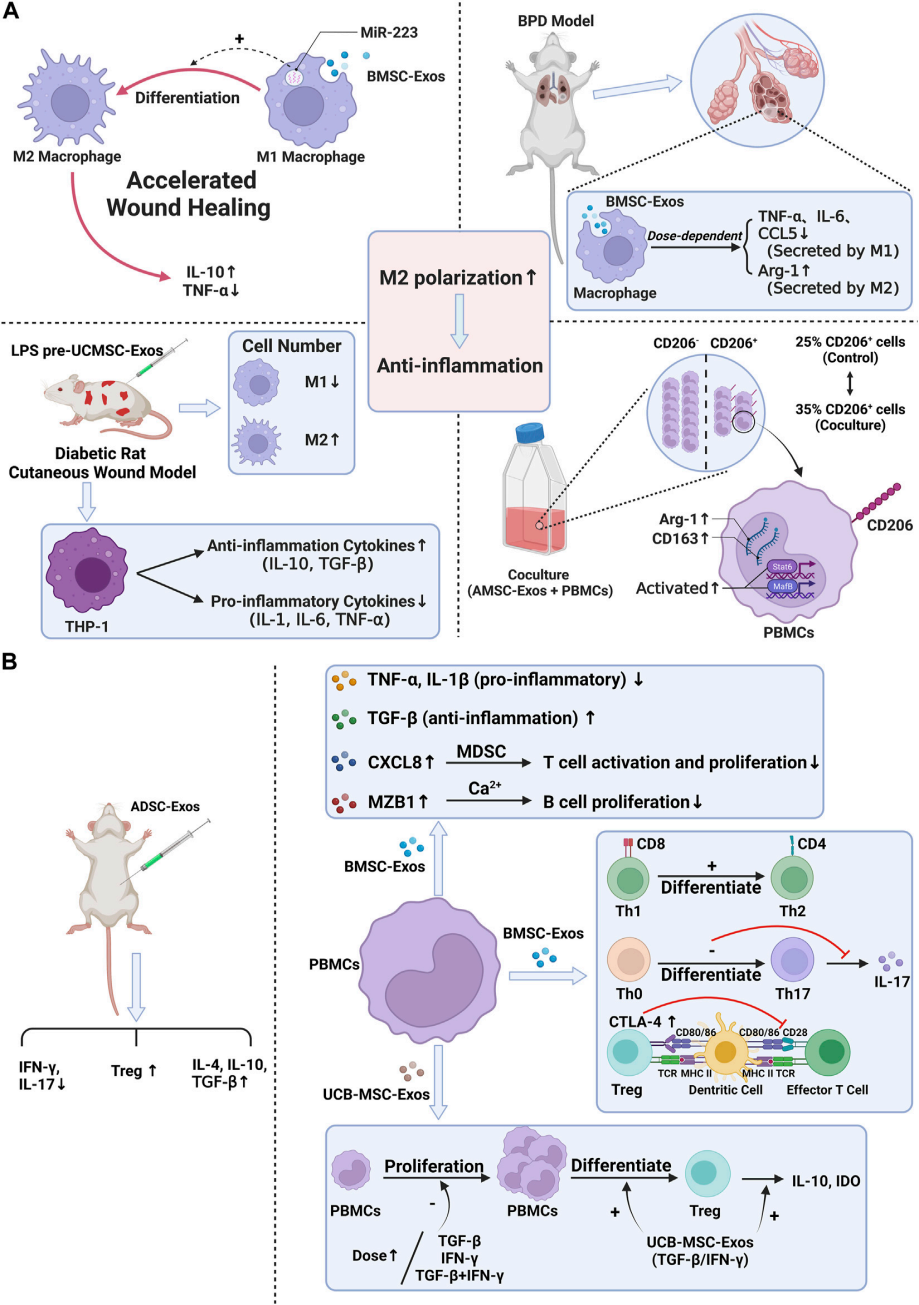
Anti-inflammation ability of stem cell exosomes. Panel **(A)**: Stem cell exosomes play anti-inflammation roles by inducing M2 macrophage polarization. BMSC-Exos induced M2 macrophage polarization through miR-223, resulting in higher IL-10 levels and lower TNF-α levels. After uptake of BMSC-Exos, the pro-inflammatory factors decreased, and the anti-inflammation factors increased. Under the induction of LPS pre-UCMSC-Exos, the M1 macrophages were decreased, while the M2 macrophages were increased. THP-1 produced more anti-inflammation cytokines and fewer pro-inflammatory cytokines. When PBMCs were cocultured with AdMSC-Exos, CD163, Arg1, and CD206-positive cells were increased. M2 macrophage–specific transcription factors were activated. Panel **(B)**: Stem cell exosomes play anti-inflammation roles by regulating the expression of cytokines, inhibiting the proliferation of PBMC, and reducing the number and function of immune cells. For T cells, after BMSC-Exos treatment, the pro-inflammatory cytokines decreased, and the anti-inflammation cytokines increased. Moreover, exosomes induced the differentiation of T cells and the higher expression of CTLA-4. UCB-MSC-Exos inhibited PBMC proliferation. More PBMCs transformed into Treg cells, and IL-10 and IDO were increased. After ADSC-Exos treatment, the number of Treg cells and the levels of IL-4, IL-10, and TGF-β were increased, while IFN-γ and IL-17 decreased. For B cells, BMSC-Exos treatment induced higher levels of CXCL8 and MZB1, which, respectively, inhibited T-cell activation and proliferation and inhibited B-cell proliferation.

To sum up, the effect of stem cell exosomes is mainly achieved by regulation of immune cells and cytokines, and the effect of exosomes may be used as an essential means to inhibit particle-induced aseptic inflammation. Therefore, applying stem cell exosomes is a new idea to prevent the aseptic loosening of prostheses in the future (Figure 3) (Table 2).

**TABLE 2:** Key variants in the anti-inflammatory pathway.

Type of immune cell	Type of exosome	Upregulated factor	Downregulated factor	Reference
Macrophages	BMSC-Exos	IL-10 and Arg-1	TNF-α	Willis et al. (2018) and He et al. (2019)
	LPS pre-UCMSC-Exos	IL-10, TGF-β, and CD163	IL-1, IL-6, and TNF-α	Ti et al. (2015)
	PBMCs cocultured with AdMSC-Exos	CD163, Arg1, CD206, Stat6, and MafB		Heo et al. (2019)
T cells	PBMCs with BMSC-Exos	CTLA-4	TNF-α, IL-1β, and IL-17	(Chen, Huang, Han, Yu, Li, Lu, et al.)
	PBMCs with UCB-MSC-Exos		PBMC	Nojehdehi et al. (2018)
	PBMC with MSC-Exos	IL-10 and IDO		Nojehdehi et al. (2018)
	ADMSC-Exos	Number of Treg cells, IL-4, IL-10, and TGF-β	IFN-γ and IL-17	Nojehdehi et al. (2018)
B cells	PBMCs with BMSC-Exos	IL-8 and MZB1		Khare et al. (2018)

## Comparison of treatments for aseptic loosening

In the face of aseptic loosening of prostheses, most of the current clinical treatments for aseptic loosening are in the experimental stage, and most of the current treatment strategies focus on the use of new materials or the adjustment of prosthesis components. Revision arthroplasty is often the ultimate measure of severe loosening. At present, the methods that have been put into research on aseptic loosening include drug therapy, gene therapy, and cell therapy.

Some drugs have been shown to prevent osteolysis, such as drugs that suppress osteoclast activity (e.g., bisphosphonates); drugs that promote osteogenesis, such as BMP; and drugs that act on inflammatory signaling pathways or cytokines, such as TNF-α antagonists. However, these drugs cannot be put into clinical use. On the one hand, these drugs are still in preclinical trials, and on the other hand, some drugs (drugs acting on cytokines) may have unknown adverse effects on other systems of the body (Smith and Schwarz, 2014). The use of drugs for the treatment of aseptic loosening still needs further clinical trials and studies.

Gene therapy is a treatment that has emerged in recent years. Ulrich-Vinther et al. (2002) investigated the use of a recombinant adeno-associated viral (RAAV) vector expressing OPG for gene therapy to construct a RAAV vector co-expressing OPG (RAAV-OPG-IRES-EGFP) and then found that OPG can effectively inhibit wear particle–induced osteoclastogenesis and osteolysis. However, this method is also in the experimental stage, and gene regulation, vector selection, and other aspects also need to be further improved.

Local therapeutic cell delivery can directly or indirectly affect osteolysis. Autologous bone grafting is a form of local cell therapy in which osteoblasts and other cells in the bone graft complex can be implanted into the bone graft to regulate the inflammatory cascade and provide autocrine and paracrine factors to support bone healing. Some researchers have used methods of local delivery of MSCs to modulate the inflammatory response and promote osteogenic differentiation and bone healing, which may be used as a potential treatment in the future (Lin et al., 2017a; Lin et al., 2019).

In contrast to the previously mentioned methods, the use of exosomes for the treatment of aseptic loosening has many advantages. Exosomes are smaller, which makes them quickly circulate *in vivo* and reach the injured site (Mendt et al., 2018). Derived from cells, exosomes are safer and lower in immunogenicity. Good membrane-bound characteristics make the contents have good biocompatibility and stability, and it is easier to cross the blood–brain barrier (Akbari and Rezaie, 2020). As a non-cellular product, exosome transplantation does not undergo rejection and harmful differentiation and malignant transformation that may occur when MSCs are transplanted (Harrell et al., 2019). Unique materials combined with stem cell exosomes have broad prospects for the treatment of aseptic loosening of the prosthesis. Korda et al. (2008) confirmed that the combination of autologous mesenchymal stem cells and allogenic bone enhanced the integration of femoral prosthesis in a sheep hemiarthroplasty model. Compared with the allograft alone treatment group, the graft healing rate in the MSC treatment group was increased, the graft absorption decreased, and the failure rate decreased (Hernigou et al., 2014). Vulcano et al. (2013) reported similar results for the reconstruction of bone defects around the acetabulum after aseptic loosening in five unrelated patients. A case report by Jäger et al. (2006)described the treatment of periacetabular osteolysis with BMP2/MSC composites. Progressive healing was reported with satisfactory results (Jäger et al., 2006). In summary, the use of exosomes for the treatment of aseptic loosening is a promising approach.

## Conclusion and foresight

In recent years, total joint replacement (TJR) has been the most cost-effective and successful surgical intervention for end-stage osteoarticular disease. However, the operation of total joint replacement has been dramatically increased and modified by international organizations, the main reason behind which is the periprosthetic osteolysis and aseptic loosening caused by TJR (Kurtz et al., 2005; Kurtz et al., 2007). Implant wear and subsequent biomaterial wear particles released into the surrounding tissue are the leading causes of periprosthetic osteolysis (Schmalzried et al., 1992). These wear particles disperse through the articular fluid along the bone–implant interface (Revell, 2008). In the tissue surrounding the prosthesis, wear debris is consumed by macrophages, activating inflammatory phenotype, secreting cytokines and chemokines (Nich et al., 2013; Pajarinen et al., 2014), and recruiting more macrophages (Lin et al., 2017b). Exosomes have tremendous therapeutic potential in related bone diseases, such as aseptic loosening of prostheses. Exogenous stem cell exosomes enhance bone binding and alleviate peri-implant osteolysis through paracrine regulation. Osteoblasts promote osteogenic mRNAs in exosomes, mediate anti-osteogenic miRNAs, and upregulate bone growth through Wnt/MAPK/PI3K-Akt pathways (Yang et al., 2020b; Zhai et al., 2020). Bone resorption is activated by RANK, tartrate-resistant acid phosphatase (TRAP), and OPG in exosomes (Inder et al., 2014; Raimondi et al., 2015). Vascular growth is closely related to bone regeneration. Although the wear debris of prosthesis blocks vascular growth, a large number of MSC exosomes contain the inclusions that stimulate vascular regeneration except for human umbilical vein endothelial cells (Zhao et al., 2020). Over-transfer of DMBT1 protein functions as a positive regulator of angiogenesis and wound healing of diabetic soft tissue. Thus, its operation in aseptic loosening of prosthesis needs further study (Chen et al., 2018). Enhancing osteogenesis and angiogenesis and suppressing osteoclast is a new idea to solve the aseptic loosening of prostheses. In summary, different stem cell exosomes play an important role in promoting osteogenesis, angiogenesis, and silencing osteoclasts and macrophage-mediated inflammation, providing a new idea for the clinical prevention and treatment of aseptic loosening of the prosthesis.

In the face of aseptic loosening of prostheses, clinical attention should be paid to prevention. Most of the current treatment strategies focus on the use of new materials or the adjustment of prosthesis components. Unique materials combined with stem cell exosomes have broad prospects for the treatment of aseptic loosening of prostheses. Korda et al. (2008) confirmed that the combination of autologous mesenchymal stem cells and allogeneic bone enhanced the integration of femoral prosthesis in the sheep hemiarthroplasty model. Compared with the allograft alone treatment group, the graft healing rate in the MSC treatment group was increased, the graft absorption decreased, and the failure rate decreased (Hernigou et al., 2014). Vulcano et al. (2013) reported similar results for the reconstruction of bone defects around the acetabulum after aseptic loosening in five unrelated patients. A case report by Jäger et al. (2006) described the treatment of periacetabular osteolysis with BMP2/MSC composites. Progressive healing was reported with satisfactory results. Although it has been less effective in alleviating the problems caused by TJR with materials, the effects of prosthesis on osteoclastogenesis and angiogenesis in patients are completely avoided. In recent years, stem cell exosomes have been widely used to regulate bone metabolism and inhibit inflammation by promoting osteogenesis and angiogenesis, having broad application prospects in tissue repair and injury prevention. Focusing on the regulation of stem cell exosomes combined with targeted drug therapy will provide more possibilities for patients to adapt to prostheses. Stem cell exosomes offer an effective treatment for bone metabolic diseases such as osteoporosis (Pajarinen et al., 2017). Exosomes from MSCs may be a promising alternative therapy based on cells (Saribas et al., 2020). Exosomes are replicated, so there is no risk of tumor formation. In addition, exosomes are much smaller than stem cells, which quickly circulate *in vivo* and reach the injured site (Mendt et al., 2018). At the same time, considering that most studies on exosomes derived from MSCs are currently in the preclinical stage, the traditional methods of exosome isolation and characterization are not effective for clinical application. The exact mechanism of MSC-derived exosomes in osteogenesis, osteoclast differentiation, angiogenesis, and inflammation remains unclear and needs further study.

Most of the current clinical treatments for aseptic loosening are in the experimental stage, and most of the current treatment strategies focus on the use of new materials or the adjustment of prosthesis components (Jäger et al., 2006; Korda et al., 2008; Vulcano et al., 2013). At present, the methods that have been put into research on aseptic loosening include drug therapy, gene therapy, and cell therapy (Hernigou et al., 2014). Some drugs have been shown to prevent osteolysis, but these drugs cannot be put into clinical use because they have not passed the clinical trial and will have other adverse effects (Jäger et al., 2006). Gene therapy has emerged in recent years. However, this method is also in the experimental stage, and gene regulation, vector selection, and other aspects also need to be further improved. Cell therapy like local delivery of MSC may serve as a future treatment for aseptic loosening (Mendt et al., 2018; Saribas et al., 2020). In contrast to the previous methods, exosomes have the merits of smaller size, greater safety, lower immunogenicity, better membrane-bound characteristics, and so on. In addition, unique materials combined with stem cell exosomes have broad prospects for the treatment of aseptic loosening of the prosthesis, which makes them a better method for treating aseptic loosening. In summary, the use of exosomes for the treatment of aseptic loosening is a promising approach.

## Glossary

AdMSC-Exos: Adipose-derived exosomes
ADSC-Exos: Adipose-derived stem cell-derived exosomes
ALP: Alkaline phosphatase
Akt: Protein kinase b
AP-1: Activator protein-1
ADSC: Adipose-derived stem cells
BMP: Bone morphogenetic protein
BMSC: Bone marrow-derived mesenchymal stem cell
BPD: Bronchopulmonary dysplasia
Arg-1: Arginase-1
CM: Conditional medium
CTHRC1: Silent information regulator 7
DMBT1: Malignant brain tumor 1
Exos: Exosomes
HA: Hydroxyapatite
HUVEC: Human umbilical vein endothelial cell
hAD-MSC: Human adipose-derived MSC
HIF-1α: Hypoxia-inducible factor-1
UCB-MSC: Human umbilical cord mesenchymal stem cell: Umbilical cord blood-derived mesenchymal stem cell
IL-1: Interleukin-LPS pre-UCMSC-Exos
MSC: Human mesenchymal stem cell
MafB: MAF BZIP transcription factor B
MAPKs: Mitogen-activated protein kinases
MDSC: Myeloid-derived suppressor cell
MZB1: Marginal zone B- and B1-cell-specific protein
OPG: Osteoclastogenesis inhibitory factor
PI3K: Activate phosphoinositol-3
RAAV: Recombinant adeno-associated viral
RANK: Receptor activator of nuclear factor κB
RANKL: Receptor activator of nuclear factor κB ligand
PBMC: Peripheral blood mononuclear cell
RUNX2: Runt-related transcription factor-2
SCI: Spinal cord injury
SIRT7: Silent information regulator 7
Smad1: Drosophila mothers against decapentaplegic protein 1
Stat6: Signal transducer and activator of transcription 6
SPRED1: Sprouty-related EVH1 domain protein 1
TNF-α: Tumor necrosis factor-α
TGF-β-1: Transform growth factor-β-1
Th17: T helper cell 17
TJR: Total joint replacement
TRAF6: Tumor necrosis factor receptor-associated factor 6
TRAP: Tartrate-resistant acid phosphatase
UCB-MSC: Human umbilical cord mesenchymal stem cell: Umbilical cord blood-derived mesenchymal stem cell
USC: Urine-derived stem cell
VEGF: Vascular endothelial growth factor.

## References

[B1] Akbari A. Rezaie J. (2020). Potential therapeutic application of mesenchymal stem cell-derived exosomes in SARS-CoV-2 pneumonia. Stem Cell Res. Ther. 11 (1), 356. 10.1186/s13287-020-01866-6 32795359 PMC7427273

[B2] Altaf H. Revell P. A. (2013). Evidence for active antigen presentation by monocyte/macrophages in response to stimulation with particles: The expression of NFκB transcription factors and costimulatory molecules. Inflammopharmacology 21 (4), 279–290. 10.1007/s10787-013-0170-z 23670535

[B3] An J. H. Park H. Song J. A. Ki K. H. Yang J. Y. Choi H. J. (2013). Transplantation of human umbilical cord blood-derived mesenchymal stem cells or their conditioned medium prevents bone loss in ovariectomized nude mice. Tissue Eng. Part A 19 (5-6), 685–696. 10.1089/ten.tea.2012.0047 23215868 PMC3568969

[B4] Bai L. Du Z. Du J. Yao W. Zhang J. Weng Z. (2018). A multifaceted coating on titanium dictates osteoimmunomodulation and osteo/angio-genesis towards ameliorative osseointegration. Biomaterials 162, 154–169. 10.1016/j.biomaterials.2018.02.010 29454274

[B5] Beckmann J. Meier M. K. Benignus C. Hecker A. Thienpont E. (2021). Contemporary knee arthroplasty: One fits all or time for diversity? Arch. Orthop. Trauma Surg. 141, 2185–2194. 10.1007/s00402-021-04042-4 34269891 PMC8595166

[B6] Berebichez-Fridman R. Gómez-García R. Granados-Montiel J. Berebichez-Fastlicht E. Olivos-Meza A. Granados J. (2017). The holy grail of orthopedic surgery: Mesenchymal stem cells-their current uses and potential applications. Stem Cells Int. 2017, 1–14. 10.1155/2017/2638305 PMC5494105 28698718

[B7] Berebichez-Fridman R. Montero-Olvera P. R. (2018). Sources and clinical applications of mesenchymal stem cells: State-of-the-art review. Sultan Qaboos Univ. Med. J. 18 (3), e264. 10.18295/squmj.2018.18.03.002 30607265 PMC6307657

[B8] Boyce B. F. Xing L. (2007). Biology of RANK, RANKL, and osteoprotegerin. Arthritis Res. Ther. 9 (1), S1. 10.1186/ar2165 17634140 PMC1924516

[B9] Buettmann E. G. McKenzie J. A. Migotsky N. Sykes D. A. Hu P. Yoneda S. (2019). VEGFA from early osteoblast lineage cells (Osterix+) is required in mice for fracture healing. J. Bone Min. Res. 34 (9), 1690–1706. 10.1002/jbmr.3755 PMC6744295 31081125

[B10] Butler J. M. Kobayashi H. Rafii S. (2010). Instructive role of the vascular niche in promoting tumour growth and tissue repair by angiocrine factors. Nat. Rev. Cancer 10 (2), 138–146. 10.1038/nrc2791 20094048 PMC2944775

[B11] Cagliani J. Grande D. Molmenti E. P. Miller E. J. Rilo H. L. R. (2017). Immunomodulation by mesenchymal stromal cells and their clinical applications. J. Stem Cell Regen. Biol. 3 (2), 1–14. 10.15436/2471-0598.17.022 PMC5667922 29104965

[B12] Chen C. Y. Rao S. S. Ren L. Hu X. K. Tan Y. J. Hu Y. (2018). Exosomal DMBT1 from human urine-derived stem cells facilitates diabetic wound repair by promoting angiogenesis. Theranostics 8 (6), 1607–1623. 10.7150/thno.22958 29556344 PMC5858170

[B13] Chen C. Y. Rao S. S. Tan Y. J. Luo M. J. Hu X. K. Yin H. (2019). Extracellular vesicles from human urine-derived stem cells prevent osteoporosis by transferring CTHRC1 and OPG. Bone Res. 7, 18. 10.1038/s41413-019-0056-9 31263627 PMC6594995

[B14] Chen W. Huang Y. Han J. Yu L. Li Y. Lu Z. (2016). Immunomodulatory effects of mesenchymal stromal cells-derived exosome. Immunol. Res. 64 (4), 831–840. 10.1007/s12026-016-8798-6 27115513

[B15] Choudhery M. S. Badowski M. Muise A. Pierce J. Harris D. T. (2014). Donor age negatively impacts adipose tissue-derived mesenchymal stem cell expansion and differentiation. J. Transl. Med. 12, 8. 10.1186/1479-5876-12-8 24397850 PMC3895760

[B16] Crotti T. N. Smith M. D. Findlay D. M. Zreiqat H. Ahern M. Weedon H. (2004). Factors regulating osteoclast formation in human tissues adjacent to peri-implant bone loss: Expression of receptor activator NFκB, RANK ligand and osteoprotegerin. Biomaterials 25 (4), 565–573. 10.1016/s0142-9612(03)00556-8 14607494

[B17] Doi H. Kitajima Y. Luo L. Yan C. Tateishi S. Ono Y. (2016). Potency of umbilical cord blood- and Wharton's jelly-derived mesenchymal stem cells for scarless wound healing. Sci. Rep. 6, 18844. 10.1038/srep18844 26728342 PMC4700425

[B18] Dorronsoro A. Robbins P. D. (2013). Regenerating the injured kidney with human umbilical cord mesenchymal stem cell-derived exosomes. Stem Cell Res. Ther. 4 (2), 39. 10.1186/scrt187 23680102 PMC3706756

[B19] Gao J. Feng Z. Wang X. Zeng M. Liu J. Han S. (2018). SIRT3/SOD2 maintains osteoblast differentiation and bone formation by regulating mitochondrial stress. Cell Death Differ. 25 (2), 229–240. 10.1038/cdd.2017.144 28914882 PMC5762839

[B20] Hankenson K. D. Dishowitz M. Gray C. Schenker M. (2011). Angiogenesis in bone regeneration. Injury 42 (6), 556–561. 10.1016/j.injury.2011.03.035 21489534 PMC3105195

[B21] Hampton C. B. Berliner Z. P. Nguyen J. T. Mendez L. Smith S. S. Joseph A. D. (2020). Aseptic loosening at the tibia in total knee arthroplasty: A function of cement mantle quality? J. Arthroplasty 35 (6S), S190–S196. 10.1016/j.arth.2020.02.028 32171492

[B22] Harrell C. R. Jovicic N. Djonov V. Arsenijevic N. Volarevic V. (2019). Mesenchymal stem cell-derived exosomes and other extracellular vesicles as new remedies in the therapy of inflammatory diseases. Cells 8 (12), 1605. 10.3390/cells8121605 PMC6952783 31835680

[B23] Hartmann E. S. Köhler M. I. Huber F. Redeker J. I. Schmitt B. Schmitt-Sody M. (2017). Factors regulating bone remodeling processes in aseptic implant loosening. J. Orthop. Res. 35 (2), 248–257. 10.1002/jor.23274 27116254

[B24] He X. Dong Z. Cao Y. Wang H. Liu S. Liao L. (2019). MSC-derived exosome promotes M2 polarization and enhances cutaneous wound healing. Stem Cells Int. 2019, 1–16. 10.1155/2019/7132708 PMC6754952 31582986

[B25] Heo J. S. Choi Y. Kim H. O. (2019). Adipose-derived mesenchymal stem cells promote M2 macrophage phenotype through exosomes. Stem Cells Int. 2019, 1–10. 10.1155/2019/7921760 PMC6875419 31781246

[B26] Hernigou P. Pariat J. Queinnec S. Homma Y. Lachaniette C. H. F. Chevallier N. (2014). Supercharging irradiated allografts with mesenchymal stem cells improves acetabular bone grafting in revision arthroplasty. Int. Orthop. 38 (9), 1913–1921. 10.1007/s00264-014-2285-2 24509980

[B27] Hodges N. A. Sussman E. M. Stegemann J. P. (2021). Aseptic and septic prosthetic joint loosening: Impact of biomaterial wear on immune cell function, inflammation, and infection. Biomaterials 278, 121127. 10.1016/j.biomaterials.2021.121127 34564034

[B28] Hu K. Olsen B. R. (2016). The roles of vascular endothelial growth factor in bone repair and regeneration. Bone 91, 30–38. 10.1016/j.bone.2016.06.013 27353702 PMC4996701

[B29] Huang J. H. Xu Y. Yin X. M. Lin F. Y. (2020). Exosomes derived from miR-126-modified MSCs promote angiogenesis and neurogenesis and attenuate apoptosis after spinal cord injury in rats. Neuroscience 424, 133–145. 10.1016/j.neuroscience.2019.10.043 31704348

[B30] Inder K. L. Ruelcke J. E. Petelin L. Moon H. Choi E. Rae J. (2014). Cavin-1/PTRF alters prostate cancer cell-derived extracellular vesicle content and internalization to attenuate extracellular vesicle-mediated osteoclastogenesis and osteoblast proliferation. J. Extracell. Vesicles 3, 23784. 10.3402/jev.v3.23784 PMC4072912 25018864

[B31] Jäger M. Emami R. Thorey F. Krauspe R. (2006). Saving implants BMP-2 application in revision total hip surgery. Int. J. Biomed. Sci. 2 (2), 187–195. 23674982 PMC3614591

[B32] Jiang L. B. Tian L. Zhang C. G. (2018). Bone marrow stem cells-derived exosomes extracted from osteoporosis patients inhibit osteogenesis via microRNA-21/SMAD7. Eur. Rev. Med. Pharmacol. Sci. 22 (19), 6221–6229. 10.26355/eurrev_201810_16028 30338786

[B33] Kalluri R. LeBleu V. S. (2020). The biology function and biomedical applications of exosomes. Science 367 (6478), eaau6977. 10.1126/science.aau6977 32029601 PMC7717626

[B34] Kern S. Eichler H. Stoeve J. Klüter H. Bieback K. (2006). Comparative analysis of mesenchymal stem cells from bone marrow, umbilical cord blood, or adipose tissue. Stem Cells 24 (5), 1294–1301. 10.1634/stemcells.2005-0342 16410387

[B35] Khare D. Or R. Resnick I. Barkatz C. Almogi-Hazan O. Avni B. (2018). Mesenchymal stromal cell-derived exosomes affect mRNA expression and function of B-lymphocytes. Front. Immunol. 9, 3053. 10.3389/fimmu.2018.03053 30622539 PMC6308164

[B36] Kim Y-J. Yoo S. M. Park H. H. Lim H. J. Kim Y-L. Lee S. (2017). Exosomes derived from human umbilical cord blood mesenchymal stem cells stimulates rejuvenation of human skin. Biochem. Biophys. Res. Commun. 493 (2), 1102–1108. 10.1016/j.bbrc.2017.09.056 28919421

[B37] Korda M. Blunn G. Goodship A. Hua J. (2008). Use of mesenchymal stem cells to enhance bone formation around revision hip replacements. J. Orthop. Res. 26 (6), 880–885. 10.1002/jor.20598 18271017

[B38] Koreny T. Tunyogi-Csapó M. Gál I. Vermes C. Jacobs J. J. Glant T. T. (2006). The role of fibroblasts and fibroblast-derived factors in periprosthetic osteolysis. Arthritis Rheum. 54 (10), 3221–3232. 10.1002/art.22134 17009257

[B39] Kurtz S. Mowat F. Ong K. Chan N. Lau E. Halpern M. (2005). Prevalence of primary and revision total hip and knee arthroplasty in the United States from 1990 through 2002. J. Bone Jt. Surg. 87 (7), 1487. 10.2106/jbjs.d.02441 15995115

[B40] Kurtz S. Ong K. Lau E. Mowat F. Halpern M. (2007). Projections of primary and revision hip and knee arthroplasty in the United States from 2005 to 2030. J. Bone Jt. Surg. 89 (4), 780–785. 10.2106/jbjs.f.00222 17403800

[B41] Kusano K. Miyaura C. Inada M. Tamura T. Ito A. Nagase H. (1998). Regulation of matrix metalloproteinases (MMP-2, -3, -9, and -13) by interleukin-1 and interleukin-6 in mouse calvaria: Association of MMP induction with bone resorption. Endocrinology 139 (3), 1338–1345. 10.1210/endo.139.3.5818 9492070

[B42] Kusumbe A. P. Ramasamy S. K. Adams R. H. (2014). Coupling of angiogenesis and osteogenesis by a specific vessel subtype in bone. Nature 507 (7492), 323–328. 10.1038/nature13145 24646994 PMC4943525

[B43] Lader C. S. Flanagan A. M. (1998). Prostaglandin E2, interleukin 1α, and tumor necrosis factor-α increase human osteoclast formation and bone resorption *in vitro**. Endocrinology 139 (7), 3157–3164. 10.1210/endo.139.7.6085 9645689

[B44] Li W. Liu Y. Zhang P. Tang Y. Zhou M. Jiang W. (2018). Tissue-engineered bone immobilized with human adipose stem cells-derived exosomes promotes bone regeneration. ACS Appl. Mat. Interfaces 10 (6), 5240–5254. 10.1021/acsami.7b17620 29359912

[B45] Li X. Liao J. Su X. Li W. Bi Z. Wang J. (2020). Human urine-derived stem cells protect against renal ischemia/reperfusion injury in a rat model via exosomal *miR-146a-5p* which targets *IRAK1* . Theranostics 10 (21), 9561–9578. 10.7150/thno.42153 32863945 PMC7449916

[B46] Lin T. Kohno Y. Huang J-F. Romero-Lopez M. Maruyama M. Ueno M. (2019). Preconditioned or IL4-secreting mesenchymal stem cells enhanced osteogenesis at different stages. Tissue Eng. Part A 25 (15-16), 1096–1103. 10.1089/ten.tea.2018.0292 30652628 PMC6686696

[B47] Lin T. Pajarinen J. Nabeshima A. Lu L. Nathan K. Yao Z. (2017). Establishment of NF-κB sensing and interleukin-4 secreting mesenchymal stromal cells as an "on-demand" drug delivery system to modulate inflammation. Cytotherapy 19 (9), 1025–1034. 10.1016/j.jcyt.2017.06.008 28739167 PMC5563472

[B48] Lin T. H. Gibon E. Loi F. Pajarinen J. Cordova L. A. Nabeshima A. (2017). Decreased osteogenesis in mesenchymal stem cells derived from the aged mouse is associated with enhanced NF-κB activity. J. Orthop. Res. 35 (2), 281–288. 10.1002/jor.23270 27105133

[B49] Liras A. (2010). Future research and therapeutic applications of human stem cells: General, regulatory, and bioethical aspects. J. Transl. Med. 8, 131. 10.1186/1479-5876-8-131 21143967 PMC3014893

[B50] Lu G. D. Cheng P. Liu T. Wang Z. (2020). BMSC-derived exosomal miR-29a promotes angiogenesis and osteogenesis. Front. Cell Dev. Biol. 8, 608521. 10.3389/fcell.2020.608521 33363169 PMC7755650

[B51] Lubberts E. van den Bersselaar L. Oppers-Walgreen B. Schwarzenberger P. Coenen-de Roo C. J. J. Kolls J. K. (2003). IL-17 promotes bone erosion in murine collagen-induced arthritis through loss of the receptor activator of NF-kappa B ligand/osteoprotegerin balance. J. Immunol. 170 (5), 2655–2662. 10.4049/jimmunol.170.5.2655 12594294

[B52] Luo G. Cheng W. He W. Wang X. Tan J. Fitzgerald M. (2010). Promotion of cutaneous wound healing by local application of mesenchymal stem cells derived from human umbilical cord blood. Wound Repair Regen. 18 (5), 506–513. 10.1111/j.1524-475x.2010.00616.x 20840520

[B53] Marie P. J. Kassem M. (2011). Osteoblasts in osteoporosis: Past, emerging, and future anabolic targets. Eur. J. Endocrinol. 165 (1), 1–10. 10.1530/eje-11-0132 21543379

[B54] Mendt M. Kamerkar S. Sugimoto H. McAndrews K. M. Wu C. C. Gagea M. (2018). Generation and testing of clinical-grade exosomes for pancreatic cancer. JCI Insight 3 (8), 99263. 10.1172/jci.insight.99263 29669940 PMC5931131

[B55] Nagy V. Penninger J. M. (2015). The RANKL-RANK story. Gerontology 61 (6), 534–542. 10.1159/000371845 25720990

[B56] Nich C. Takakubo Y. Pajarinen J. Ainola M. Salem A. Sillat T. (2013). Macrophages-Key cells in the response to wear debris from joint replacements. J. Biomed. Mat. Res. A 101 (10), 3033–3045. 10.1002/jbm.a.34599 PMC3775910 23568608

[B57] Nojehdehi S. Soudi S. Hesampour A. Rasouli S. Soleimani M. Hashemi S. M. (2018). Immunomodulatory effects of mesenchymal stem cell-derived exosomes on experimental type-1 autoimmune diabetes. J. Cell. Biochem. 119 (11), 9433–9443. 10.1002/jcb.27260 30074271

[B58] Olsen J. J. Pohl S. Deshmukh A. Visweswaran M. Ward N. C. Arfuso F. (2017). The role of Wnt signalling in angiogenesis. Clin. Biochem. Rev. 38 (3), 131–142. 29332977 PMC5759160

[B59] Pajarinen J. Lin T. H. Nabeshima A. Jamsen E. Lu L. Nathan K. (2017). Mesenchymal stem cells in the aseptic loosening of total joint replacements. J. Biomed. Mat. Res. A 105 (4), 1195–1207. 10.1002/jbm.a.35978 PMC5531266 27977880

[B60] Pajarinen J. Lin T. H. Sato T. Yao Z. Goodman S. B. (2014). Interaction of materials and biology in total joint replacement - successes, challenges and future directions. J. Mat. Chem. B 2 (41), 7094–7108. 10.1039/c4tb01005a PMC4273175 25541591

[B61] Park J. H. Lee N. K. Lee S. Y. (2017). Current understanding of RANK signaling in osteoclast differentiation and maturation. Mol. Cells 40 (10), 706–713. 10.14348/molcells.2017.0225 29047262 PMC5682248

[B62] Qin Y. Sun R. Wu C. Wang L. Zhang C. (2016). Exosome: A novel approach to stimulate bone regeneration through regulation of osteogenesis and angiogenesis. Int. J. Mol. Sci. 17 (5), 712. 10.3390/ijms17050712 PMC4881534 27213355

[B63] Rachner T. D. Khosla S. Hofbauer L. C. (2011). Osteoporosis: Now and the future. Lancet 377 (9773), 1276–1287. 10.1016/s0140-6736(10)62349-5 21450337 PMC3555696

[B64] Raimondi L. De Luca A. Amodio N. Manno M. Raccosta S. Taverna S. (2015). Involvement of multiple myeloma cell-derived exosomes in osteoclast differentiation. Oncotarget 6 (15), 13772–13789. 10.18632/oncotarget.3830 25944696 PMC4537049

[B65] Red-Horse K. Crawford Y. Shojaei F. Ferrara N. (2007). Endothelium-microenvironment interactions in the developing embryo and in the adult. Dev. Cell 12 (2), 181–194. 10.1016/j.devcel.2007.01.013 17276337

[B66] Ren L. Song Z. J. Cai Q. W. Chen R. x. Zou Y. Fu Q. (2019). Adipose mesenchymal stem cell-derived exosomes ameliorate hypoxia/serum deprivation-induced osteocyte apoptosis and osteocyte-mediated osteoclastogenesis *in vitro* . Biochem. Biophys. Res. Commun. 508 (1), 138–144. 10.1016/j.bbrc.2018.11.109 30473217

[B67] Revell P. A. (2008). The combined role of wear particles, macrophages and lymphocytes in the loosening of total joint prostheses. J. R. Soc. Interface 5 (28), 1263–1278. 10.1098/rsif.2008.0142 18647740 PMC2607446

[B68] Saribas G. S. Ozogul C. Tiryaki M. Alpaslan Pinarli F. Hamdemir Kilic S. (2020). Effects of uterus derived mesenchymal stem cells and their exosomes on asherman's syndrome. Acta Histochem. 122 (1), 151465. 10.1016/j.acthis.2019.151465 31776004

[B69] Schmalzried T. P. Jasty M. Harris W. H. (1992). Periprosthetic bone loss in total hip arthroplasty. Polyethylene wear debris and the concept of the effective joint space. J. Bone Jt. Surg. 74 (6), 849–863. 10.2106/00004623-199274060-00006 1634575

[B70] Shi Y. Yang Y. Guo Q. Gao Q. Ding Y. Wang H. (2019). Exosomes derived from human umbilical cord mesenchymal stem cells promote fibroblast-to-myofibroblast differentiation in inflammatory environments and benefit cardioprotective effects. Stem Cells Dev. 28 (12), 799–811. 10.1089/scd.2018.0242 30896296

[B71] Smith R. L. Schwarz E. M. (2014). Are biologic treatments a potential approach to wear- and corrosion-related problems? Clin. Orthop. Relat. Res. 472 (12), 3740–3746. 10.1007/s11999-014-3765-9 24993143 PMC4397762

[B72] Szczesiul J. Bielecki M. (2021). A review of total hip arthroplasty comparison in FNF and OA patients. Adv. Orthop. 2021, 1–6. 10.1155/2021/5563500 PMC8463253 34567807

[B73] Tashiro Y. Nishida C. Sato-Kusubata K. Ohki-Koizumi M. Ishihara M. Sato A. (2012). Inhibition of PAI-1 induces neutrophil-driven neoangiogenesis and promotes tissue regeneration via production of angiocrine factors in mice. Blood 119 (26), 6382–6393. 10.1182/blood-2011-12-399659 22573404

[B74] Ti D. Hao H. Tong C. Liu J. Dong L. Zheng J. (2015). LPS-preconditioned mesenchymal stromal cells modify macrophage polarization for resolution of chronic inflammation via exosome-shuttled let-7b. J. Transl. Med. 13, 308. 10.1186/s12967-015-0642-6 26386558 PMC4575470

[B75] Tofiño-Vian M. Guillén M. I. Pérez Del Caz M. D. Castejón M. A. Alcaraz M. J. (2017). Extracellular vesicles from adipose-derived mesenchymal stem cells downregulate senescence features in osteoarthritic osteoblasts. Oxid. Med. Cell. Longev. 2017, 1–12. 10.1155/2017/7197598 PMC5694590 29230269

[B76] Ulrich-Vinther M. Carmody E. E. Goater J. J. S balle K. O'Keefe R. J. Schwarz E. M. (2002). Recombinant adeno-associated virus-mediated osteoprotegerin gene therapy inhibits wear debris-induced osteolysis. J. Bone Jt. Surg. 84 (8), 1405–1412. 10.2106/00004623-200208000-00017 12177271

[B77] Vermes C. Glant T. T. Hallab N. J. Fritz E. A. Roebuck K. A. Jacobs J. J. (2001). The potential role of the osteoblast in the development of periprosthetic osteolysis: Review of *in vitro* osteoblast responses to wear debris, corrosion products, and cytokines and growth factors. J. Arthroplasty 16 (81), 95–100. 10.1054/arth.2001.28719 11742458

[B78] Vulcano E. Murena L. Falvo D. A. Baj A. Toniolo A. Cherubino P. (2013). Bone marrow aspirate and bone allograft to treat acetabular bone defects in revision total hip arthroplasty: Preliminary report. Eur. Rev. Med. Pharmacol. Sci. 17 (16), 2240–2249. 23893192

[B79] Wang L. Jia P. Shan Y. Hao Y. Wang X. Jiang Y. (2017). Synergistic protection of bone vasculature and bone mass by desferrioxamine in osteoporotic mice. Mol. Med. Rep. 16 (5), 6642–6649. 10.3892/mmr.2017.7451 28901524 PMC5865796

[B80] Wang Y. Wan C. Deng L. Liu X. Cao X. Gilbert S. R. (2007). The hypoxia-inducible factor alpha pathway couples angiogenesis to osteogenesis during skeletal development. J. Clin. Invest. 117 (6), 1616–1626. 10.1172/jci31581 17549257 PMC1878533

[B81] Willis G. R. Fernandez-Gonzalez A. Anastas J. Vitali S. H. Liu X. Ericsson M. (2018). Mesenchymal stromal cell exosomes ameliorate experimental bronchopulmonary dysplasia and restore lung function through macrophage immunomodulation. Am. J. Respir. Crit. Care Med. 197 (1), 104–116. 10.1164/rccm.201705-0925oc 28853608 PMC5765387

[B82] Wu C. Chen L. Huang Y-Z. Huang Y. Parolini O. Zhong Q. (2018). Comparison of the proliferation and differentiation potential of human urine-placenta decidua basalis-and bone marrow-derived stem cells. Stem Cells Int. 2018, 1–11. 10.1155/2018/7131532 PMC6311712 30651734

[B83] Wu X. Pang L. Lei W. Lu W. Li J. Li Z. (2010). Inhibition of Sca-1-positive skeletal stem cell recruitment by alendronate blunts the anabolic effects of parathyroid hormone on bone remodeling. Cell Stem Cell 7 (5), 571–580. 10.1016/j.stem.2010.09.012 21040899 PMC4084813

[B84] Xu R. Shen X. Si Y. Fu Y. Zhu W. Xiao T. (2018). MicroRNA-31a-5p from aging BMSCs links bone formation and resorption in the aged bone marrow microenvironment. Aging Cell 17 (4), e12794. 10.1111/acel.12794 29896785 PMC6052401

[B85] Xue C. Shen Y. Li X. Li B. Zhao S. Gu J. (2018). Exosomes derived from hypoxia-treated human adipose mesenchymal stem cells enhance angiogenesis through the PKA signaling pathway. Stem Cells Dev. 27 (7), 456–465. 10.1089/scd.2017.0296 29415626

[B86] Yang J. Chen Z. Pan D. Li H. Shen J. (2020). Umbilical cord-derived mesenchymal stem cell-derived exosomes combined pluronic F127 hydrogel promote chronic diabetic wound healing and complete skin regeneration. Int. J. Nanomedicine 15, 5911–5926. 10.2147/ijn.s249129 32848396 PMC7429232

[B87] Yang S. Guo S. Tong S. Sun X. (2020). Exosomal miR-130a-3p regulates osteogenic differentiation of Human Adipose-Derived stem cells through mediating SIRT7/Wnt/β-catenin axis. Cell Prolif. 53 (10), e12890. 10.1111/cpr.12890 32808361 PMC7574877

[B88] Yang S. Zhu B. Yin P. Zhao L. Wang Y. Fu Z. (2020). Integration of human umbilical cord mesenchymal stem cells-derived exosomes with hydroxyapatite-embedded hyaluronic acid-alginate hydrogel for bone regeneration. ACS Biomater. Sci. Eng. 6 (3), 1590–1602. 10.1021/acsbiomaterials.9b01363 33455380

[B89] Yuan F. L. Wu Q. Y. Miao Z. N. Xu M. H. Xu R. S. Jiang D. L. (2018). Osteoclast-derived extracellular vesicles: Novel regulators of osteoclastogenesis and osteoclast-osteoblasts communication in bone remodeling. Front. Physiol. 9, 628. 10.3389/fphys.2018.00628 29910740 PMC5992398

[B90] Zhai M. Zhu Y. Yang M. Mao C. (2020). Human mesenchymal stem cell derived exosomes enhance cell-free bone regeneration by altering their miRNAs profiles. Adv. Sci. (Weinh). 7 (19), 2001334. 10.1002/advs.202001334 33042751 PMC7539212

[B91] Zhang L. Jiao G. Ren S. Zhang X. Li C. Wu W. (2020). Exosomes from bone marrow mesenchymal stem cells enhance fracture healing through the promotion of osteogenesis and angiogenesis in a rat model of nonunion. Stem Cell Res. Ther. 11 (1), 38. 10.1186/s13287-020-1562-9 31992369 PMC6986095

[B92] Zhang Q. Fu L. Liang Y. Guo Z. Wang L. Ma C. (2018). Exosomes originating from MSCs stimulated with TGF-β and IFN-γ promote Treg differentiation. J. Cell. Physiol. 233 (9), 6832–6840. 10.1002/jcp.26436 29336475 PMC13482522

[B93] Zhang Y. Zhang P. Gao X. Chang L. Chen Z. Mei X. (2021). Preparation of exosomes encapsulated nanohydrogel for accelerating wound healing of diabetic rats by promoting angiogenesis. Mater. Sci. Eng. C 120, 111671. 10.1016/j.msec.2020.111671 33545836

[B94] Zhao G. Ge Y. Zhang C. Zhang L. Xu J. Qi L. (2020). Progress of mesenchymal stem cell-derived exosomes in tissue repair. Curr. Pharm. Des. 26 (17), 2022–2037. 10.2174/1381612826666200420144805 32310043

